# Knowledge, Attitudes and Perceptions of Secondary School Teenagers towards HIV Transmission and Prevention in Rural and Urban Areas of Central Uganda

**DOI:** 10.4236/health.2016.810097

**Published:** 2016-07-14

**Authors:** Annamaria Rukundo, Mathias M. Muwonge, Danny Mugisha, Dickens Aturwanaho, Arabat Kasangaki, Godfrey S. Bbosa

**Affiliations:** 1 School of Medicine, Makerere University College of Health Sciences, Kampala, Uganda; 2 Department of Dentistry, Makerere University College of Health Sciences, Kampala, Uganda; 3 Department of Pharmacology & Therapeutics, Makerere University College of Health Sciences, Kampala, Uganda

**Keywords:** Knowledge, Attitudes, Perceptions, HIV Transmission & Prevention, Secondary School Teenagers

## Abstract

HIV/AIDS has remained a challenge in Uganda among adolescent despite the ABC strategy used globally to prevent HIV infection. The study assessed the knowledge, attitudes and perceptions of secondary school teenagers towards HIV transmission and prevention in rural and urban schools of central Uganda. A cross sectional study using self-administered questionnaires and structured interviews was used to collect data from adolescents in secondary schools in Kampala and Buikwe districts. Eight schools were randomly selected with 4 schools in each district. A total of 245 students from schools were recruited in the study with 120 and 125 students from urban Kampala and rural Buikwe district schools respectively. Data were analyzed using SPSS version 11. The results were expressed as percentages in a 2 × 2 tables. The mean age of the participants was 15.9 ± 2.5 years. Results showed that 95.1% participants had knowledge on HIV/AIDS in both urban and rural schools and 27.4% knew all the modes of HIV transmission. About 83.7% knew the ABC strategy for HIV prevention and 37.6% would talk about HIV/AIDS mainly with friends. For HIV cure, 62.0% of study participants reported non-cure and 24.9% were not sure. The remaining 13.1% of the study participants in both urban and rural schools reported that HIV can be cured. And the modes of curing HIV that were mentioned by participants included spiritual healing, transmitting it to others through sexual intercourse and that antiretroviral (ARVs) drugs can cure it as well as that it can be cured abroad. About 65.7% of participants reported recognition of one with HIV/ AIDS and by having red lips, being sickly; weight loss, skin rash and being very rich were mentioned. About 39.2% of the study participants mentioned that they cannot get infected with HIV and can’t contract HIV at all and 18.4% believed that chances of getting HIV infection were high. On perception and attitude on condoms and their use, participants reported that it is a sign of mistrust, reduces sexual pleasures and they are embarrassing to buy. Majority of the participants in both urban and rural schools had some knowledge on HIV/AIDS and the ABC strategy for HIV prevention. However, there was a knowledge gap on the various modes of HIV transmission and prevention. There was misconception of the participants on HIV/AIDS cure, condom use and on the chances of contracting HIV. About the source of HIV/AIDS information, majority of the participants were getting information on HIV/AIDS from friends of which some information was misleading. This false information could be the reason for the increased HIV prevalence reported among the adolescents in the schools. Generally, participants had some knowledge on HIV/AIDS though they had knowledge gap on HIV transmission and prevention.

## 1. Introduction

Human immunodeficiency virus (HIV) and acquired immunodeficiency syndrome (HIV/AIDS) remain one of the most serious global challenges the world is facing today and most especially in the sub-Saharan African countries [1] [2]. Globally, it was reported that by end of June 2015, about 36.9 million people were living with HIV and of these; 15.8 million people were accessing antiretroviral therapy. And about 1.2 million people had died of HIV/AIDS-related illnesses in the same period [1] [2]. Of the estimated global population living with HIV, 25.8 million people were reported to occur in sub-Saharan Africa with women accounting for more than 50% of the infected people [1]–[3]. About 2 million people were reported to have become newly infected with HIV globally by end of June 2015 and of these, more than 95% occurred in developing countries with about 1.4 million people reported to occur in the worst hit sub-Saharan African nations [1]–[3].

Globally, the most vulnerable group of individuals to HIV infection are reported to be the youth in the reproductive age group of 15 – 24 years with adolescents contributing to a large percentage [4] [5]. In sub-Saharan Africa, an estimated 3.8 million youth are living with HIV/AIDS and this contributes to 76% of the world’s HIV positive youth [4] [5]. The youth are reported to contribute to about 41% of all new HIV infections with an estimated 2500 young people getting infected daily [4] [5]. The young women contribute to about 60% of the youth living with HIV [4] [5]. HIV is the leading cause of death of women in reproductive age in low- and middle-income countries, especially in the sub-Saharan Countries [6] [7].

In Uganda, the burden of HIV is continuing to be a national problem especially among the youth in adolescent age group that are both schooling and the non-schooling. The HIV prevalence in Ugandan general population following the two AIDS Indicator Surveys showed that it had increased from 6.4% in 2004/5 to 7.4% in 2012/ 2013 [8] [9]. However, the HIV prevalence in the country has been reported to vary by social dynamics, geographical location and in urban or rural areas [9]–[11]. According to published reports, the HIV prevalence has been reported to be higher in the urban areas (8.7%) as compared to the rural areas (7.1%) [9]–[11]. The same report also shows that the HIV prevalence is more in women (8.3%) as compared to men (6.1%) and a similar trend is observed among the never married, married, divorced and widowed. The HIV prevalence is reported to be high among women in the age group of 15 – 39 years as compared to men except for the age group of 15 – 17 years where there is no gender difference [9]–[12]. And the prevalence was reported to peak at the age of 35 – 39 years for women (12.1%) and 40 – 44 years for men (11.3%) respectively [9]–[12].

The high HIV prevalence among the female adolescents has been attributed to the lack of power to negotiate with their male counterparts on the safe sexual matters especially if there is a cross-generation relationship [13]–[16]. Poverty among the young women or adolescents also contributes to the high HIV prevalence since sex may have financial attachment [15] [16]. Also, the youth especially students may have multiple sexual partners; some of whom experience early life sexual activities thus predisposing them to HIV infection. In Uganda, almost 25% of the people who are living with HIV/AIDS are a part of the education system that includes students and staff [17], and therefore may be strongly associated with increased transmission of HIV virus among themselves. The peer pressure, misconceptions about sexuality, influence of mass media and increased internet access which predisposes the youth to pornographic materials that arouse make them eventually engage in unsafe sexual activities thus predisposing them to HIV infection. And the exposure is more common in urban areas and schools as opposed to rural areas where the burden of HIV/AIDS is reported to be high especially among adolescents [11] [18] [19], despite of the rigorous campaigns of the ABC HIV prevention strategy that saw a reduction of HIV prevalence from 30% in 1990’s to 6.4% in 2004/2005, where it stabilized for some time until 2014 after which it rose to 7.3% [1] [8] [19].

Whereas, the ABC strategy (abstinence, being faithful and condom use), tremendously reduced the HIV prevalence from 30% in early 1990’s to nearly 6.4% by 2004/2005 [20]–[22], but in 2011, the HIV prevalence rate slightly increased to 7.3% possibly because of the behavioral change, knowledge gap on HIV and the socio-economic hardship experienced in the Ugandan population [13] [20] [21]. Research globally reports that comprehensive school and community-based HIV prevention programs, which include providing correct information on abstinence, condoms and their correct use, monogamy/partner reduction, and promoting virginity programmes have led to a reduction in the HIV prevalence and improved the exposure to risky behaviors among young people [5] [23]–[27]. However, despite of all these efforts that have been instituted in the various communities of Uganda, the HIV prevalence among the youth especially in the adolescent age is on increase and hence becoming a national public health concern. The study therefore sought to assess knowledge, attitudes and perceptions of secondary school teenagers towards HIV transmission and prevention in rural and urban schools of central Uganda.

## 2. Methods

### 2.1. Study Design

A cross-sectional study using self-administered questionnaire and structured interviews was carried out between October and November 2014, among secondary school adolescent students in both urban and rural schools of central Uganda. The essential features of the study included: 1) sampling of the secondary schools located in the district 2) Sampling of the adolescents that participated in the study 3) Data collection using a self-administered questionnaire and structured interview and 4) Conducting analysis of the collected data.

### 2.2. Study Setting

Study was conducted in eight secondary schools in Kampala and Buikwe district in Uganda. An equal number of schools (4 schools in each) were randomly selected from Kampala city, the capital and largest city in Uganda that represented urban district and Buikwe district to represent rural areas. Kampala district was chosen purposively and for convenience while Buikwe district was chosen purposively and for logistical reasons. Buikwe district is located in central Uganda and is bordered by Kayunga District to the north, Jinja district to the east, Buvuma district to the southeast, the Republic of Tanzania to the south and Mukono district to the east. It was created by Act of Parliament and commenced operations on 1 July 2009.

### 2.3. Study Population and Sampling Procedures

The target population was the adolescents, both male and female students in rural and urban areas of central Uganda. The accessible population was the sample size of students in the selected secondary schools. Sampling was done using multistage cluster sampling procedure. The schools were stratified in each selected district. A total of eight secondary schools were considered, with each selected district having 4 schools. A total of 245 participants were recruited into the study with 120 from urban schools of Kampala city and 125 participants from rural schools of Buikwe district. The sample size was adequate with an assumption of 80% power and an estimated error margin of 0.05. Using the Uganda Ministry of Education and Sports list of schools in Kampala and Buikwe district, the secondary schools were randomly selected. Four schools represented each district and of these, half were government sponsored and the rest were private schools. The sample size for each school was determined by proportionate sampling. In each school, a students’ list was obtained from which participants were randomly selected. The schools consisted of a total population of 4392 students with 2126 males and 2266 females. Within the selected schools, the participants in all classes aged between 10 to 19 years were recruited, consented/assented before the questionnaire with structured interviews were administered to them.

### 2.4. Selection Criteria of the Study Participants

The participants were both male and female students in the secondary schools within the age bracket of 10 – 19 years. Participants with the age group of 10 – 17 years had to give informed consent and assent and those between 18 – 19 years gave consent.

### 2.5. Data Collection Tool and Procedures

Primary data were collected using a pre-tested self-administered questionnaire with structured interviews. The questionnaire was designed after considering recommendations from previous researchers on Theory of Planned Behaviour (TPB) and the ecological perspectives [28]. The questionnaire had both closed and open ended questions which assessed knowledge of the students about HIV/AIDS and their attitudes towards HIV transmission, prevention and condom use. The questionnaire was pre-tested on third year medical students at Makerere University College of Health Sciences. The questionnaire collected information related to sociodemographic variables, knowledge, attitude and practices on HIV transmission and prevention. The questions were presented in three format: yes or no, Likert scale questions with responses ranging from three categories (agree, uncertain, disagree) or five categories (totally agree, agree, uncertain, disagree, and totally disagree) responses [29] [30]. The questionnaire was administered within the classroom by a trained research assistant. Research assistants were introduced to students by a teacher who later on was asked to leave the classroom to give students more freedom and privacy in their responses. Participation was voluntary and the questionnaire was anonymously filled. Participants had an opportunity to withdraw at any point without being asked any questions. The questionnaire was in English since it the language of communication in the schools and any question raised by the participants was taken care of by the research assistant.

### 2.6. Study Variables and Measures

Knowledge on HIV/AIDS was measured using the participants’ views on HIV/AIDS transmission and prevention. Correct responses to these questions were analyzed and an average percentage was computed. A percentage greater than 60% represented good knowledge about HIV/AIDS. Attitudes on HIV transmission, prevention and condom use were assessed. The background and demographic characteristics included age, residence and gender, which was a dichotomous measure such as female = 0 and male = 1. Dummy variables for religion were created based on the following categories; catholic, protestant, Pentecostal, Muslim and other religions. Also residence was a dichotomous measure (urban student = 0, rural student = 1).

### 2.7. Data Quality Control

Before the study was conducted in the selected schools, questionnaires were pre-tested. Each participant responded to the questionnaire using a structured interview with the assistance of a research assistant. Adequate spacing was ensured to promote confidentiality. Questionnaires were screened, on each day for incomplete information and consistency. The principal investigator collected all the questionnaires, stored them under lock and key and personally supervised the entry of the data into a computer which was pass-worded. Data entry was done using Epi-data entry sheets created using logic checks. Double data entry was done using two separate data entrants. The two entry sheets were compared for consistency and analyzed using SPSS and any discrepancies was resolved by referring to source document.

### 2.8. Data Analysis Plan

Each response to question in the questionnaire was coded and then entered into the epidata tool, version 3.1. The data was exported and analyzed using Statistical Package for Social Scientists (SPSS version 11). The frequencies and percentage response for each question was computed and the data was transformed into a 2 × 2 table.

### 2.9. Ethical Consideration

Ethical clearance and approval for study was obtained from the School of Medicine Research and Ethics Committee (SOMREC) at Makerere University College of Health Sciences and from the Uganda National Council of Science and Technology (UNCST). For participants in the age group 10 to 17 years, assent was sought from the parents/guardians and then consent was obtained from the student. For mature and emancipated minors, informed consent was independently sought from them. For the age group of 18 to 19 years, consent was sought from the students themselves. Confidentiality of information was ensured by the principal investigator during and after the study.

## 3. Results

The study assessed the knowledge, attitudes and perceptions of secondary school teenagers towards HIV transmission and prevention in rural and urban schools of central Uganda. A total of 245 secondary participants were recruited in the study with 120 and 125 participants from urban Kampala schools and rural Buikwe schools respectively. The ratio of males to females was 1:1.

Demographic characteristics of the participants show that majority were in the age range of 15 – 18 years. The mean total age of the participants was 15.9 ± 2.5 years and for the rural and urban participants, it was 15.4 ± 2.1 years and 15.7 ± 1.8 years respectively. There was no gender difference in the place of residence whether urban or rural areas. Majority of the participants were Catholics, Anglicans and Muslims (Table 1).

On the knowledge about HIV/AIDS, 95.1%, (n = 233/245) of the study participants were knowledgeable and 4.9% (n = 12/245) were not. About 49.0% (n = 120/125) of the study participants in rural schools were knowledgeable about HIV/ AIDS as compared to the 46.1% (n = 113/120) study participants in urban schools (Table 2).

Knowledge about HIV transmission, about 27.4% (n = 67/245) of the total participants knew all the established modes of HIV transmission with 14.3% (n = 35/120) and 13.1% (n = 32/125) from urban and rural schools respectively. The sexual intercourse was the most mentioned mode HIV transmission (32.3%, n = 79/245) of the study participants with 18.0% (n = 44/120) and 14.3% (n = 35/125) from urban and rural schools respectively. Blood transfusion (6.1%, n = 15/245) with 2.5% (n = 120) and 3.7% (n = 9/125) from urban and rural schools respectively was mentioned. The sharing of sharp objects with an infected person (4.4%, n = 11/245) with 2.4% (n = 6/120) and 2.0% (n = 5/125) from urban and rural schools respectively was mentioned. The least mode of HIV transmission mentioned was oral sex or contact through wounds with an infected person (2.4%, n = 6/245) with 0.4% (n = 1/120) and 2.0% (n = 5/125) from urban and rural schools respectively (Table 3).

On the HIV prevention strategy, about 83.7% (n = 205/245) of the study participants were knowledgeable on the ABC HIV prevention strategy with rural schools more informed (47.4%, n = 116/125) as compared to 36.3% (n = 89/120) for urban schools. About 16.3% (n = 40/245) with 12.7% (n = 31/120) and 3.6% (n = 9/125) from urban and rural schools respectively were not knowledgeable about the ABC HIV prevention strategy (Table 4).

The age, at which the participants first heard about HIV/AIDS information, Table 5, shows that participants first got the information on HIV/AIDS as early as 4 – 8 years and majority from age of 9 – 12 years (55%, 136/245) with 24.1% (n = 59/120) and 31.4% (n = 77/125) for urban and rural schools respectively. The findings show that 37.6% (n = 92/245) of the study participants got HIV/AIDS information in both urban and rural schools from the friends (Table 6).

Responses on whether HIV/AIDS is curable, about 62.0% (n = 152/245) of the study participants with 30.2% (n = 74/120) and 31.8% (n = 78/125) in urban and rural areas respectively reported that there is no cure for HIV/AIDS. Whereas 13.1% (n = 32/245) reported that there is cure for the diseases and 24.9% (n = 61/245) reported not sure (Table 7). And for those who reported that it is curable, about 50.0% (n = 16/32) of the study participants in both urban and rural schools reported that antiretroviral (ARVs) drugs can cure it. About 28.2% (n = 9/32) with 21.9% (n = 7/32) and 6.3% (n = 2/32) in both urban and rural schools respectively reported that it can be cured abroad. The results also show that 15.6% (n = 5/32) with 12.5% (n = 4/32) and 3.1% (n = 1/32) in both urban and rural schools respectively reported that it can be spiritually cured. The participants in urban schools also reported that it can be cured if transmitted to other people (6.2%, n = 2/32) (Table 8).

On whether, one can physically recognize if someone has HIV/AIDS. About 54.7% (n = 134/245) participants with 24.5% (n = 60/120) and 30.2% (n = 74/125) in urban and rural areas respectively mentioned “SOMETIMES YES”. And 34.3% (n = 84/245) with 19.2% (n = 47/120) and 15.1% (n = 37/125) in urban and rural areas respectively mentioned that it is impossible to physically recognize if someone has HIV/AIDS (Table 9). About 11.0% (n = 27/245) with 5.3% (n = 13/120) and 5.7% (n = 14/125) in urban and rural areas respectively mentioned that it is always possible to physically recognize if someone has HIV/AIDS. For those who reported that they can physically recognize if someone has HIV/AIDS, 33.5% (n = 54/161) of the participants with 20.5% (n = 33/161) and 13.0% (n = 21/161) in urban and rural areas respectively mentioned that such individuals are always sickly. About 31.7% (n = 51/161) with 13.7% (n = 22/161) and 18.0% (n = 29/161) in urban and rural areas respectively mentioned weight loss. And 24.8% (n = 40/161) of the study participants with 8.7% (n = 14/161) and 16.1% (n = 26/161) in urban and rural areas respectively mentioned red lips as a sign of HIV/AIDS. About 9.4% (n = 15/161) with 1.9% (n = 3/161) and 7.5% (n = 12/161) in urban and rural areas respectively mentioned skin rash and 0.6% (n = 1/161) participant in urban school reported that such individuals are very rich (Table 10).

On the possibility of getting HIV infection, about 39.2% (n = 96/245) study participants with 20.4% (n = 50/120) and 18.8% (n = 46/125) in urban and rural areas respectively mentioned that it is impossible to get HIV infection. About 31.0% (n = 76/245) with 15.5% (n = 38/120) and 15.5% (n = 38/125) in urban and rural areas respectively mentioned that the chance of getting HIV infection is minimal. About 18.4% (n = 45/245) of the study participants with 8.6% (n = 21/120) and 9.8% (n = 24/125) in urban and rural areas respectively mentioned that the chance of getting HIV infection is high and 11.4% (n = 28/245) with 4.5% (n = 11/120) and 6.9% (n = 17/125) in urban and rural areas respectively mentioned that the chance of getting HIV infection is moderate (Table 11).

On the perception and attitude towards condoms and condom use, about 41.6% (n = 102/245) of the study participants with 20.4% (n = 50/120) and 21.2% (n = 52/125) in urban and rural areas respectively reported that condom use reduces sexual pleasure.

About 34.3% (n = 84/245) of the study participants with 18.0% (n = 44/120) and 16.3% (n = 40/125) in urban and rural areas respectively mentioned that using the condoms is a sign of mistrust. About 18.8% (n = 46/245) participants with 9.8% (n = 24/120) and 9.0% (n = 22/125) in urban and rural areas respectively reported that condoms are embarrassing to buy. In rural schools only, participants mentioned that condom use reduces sexual pleasure and it is a sign of mistrust (1.2%, n = 3/245). And 0.8% (n = 2/245) of the participants mentioned that condom use, reduces sexual pleasure and are embarrassing to buy and 0.4% (n = 1/245) reported that condom use is a sign of mistrust and are embarrassing to buy (Table 12).

## 4. Discussion

### 4.1. Demographic Characteristics

The study assessed the knowledge, attitudes and perceptions of secondary school teenagers towards HIV transmission and prevention in rural and urban areas of central Uganda. In this study, 51.0% (n = 125/245) of the participants were from rural schools and of the total participants, 55.5% (n = 136/245) were females. There was no difference in the gender distribution of male and female participants in the study groups. Most of the participants were residing in rural areas (51.0%) in comparison to urban areas (49.0%). The results show that all religious affiliations were represented in the study groups, with majority being Anglicans (31.5%) followed by Catholics (28.6%) and then Muslims (21.3%). The age distribution of the participants varied from 12 years to above 18 years with the majority in the range of 16 years (17.5%) and ≥18 years (17.1%) (Table 1). The mean age of the participants was 15.9 ± 2.5 years and for the rural and urban participants, it was 15.4 ± 2.1 and 15.7 ± 1.8 years respectively. Previous reports show that this age group is more vulnerable to HIV infection and that HIV prevalence is reported to be higher among the youth including the adolescents with the young women most affected as compared to the men except for the age group of 15 – 17 years where there was no gender difference [1] [8] [10] [12].

### 4.2. Knowledge about HIV/AIDS by Participants in the Urban and Rural Schools

The findings of the study showed that, generally there was an excellent awareness of the HIV/AIDS among the participants (95.1%, n = 233/245) as compared to the 4.9% (n = 12/245) participants who were not knowledgeable. Majority of the participants who were knowledgeable about HIV/AIDS were from rural schools (49.0%; n = 120/125) as compared to the 46.1% (n = 113/120) in urban schools. The lack of knowledge about HIV/AIDS from the study were mainly from urban schools 2.9% (n = 7/120) as compared to the rural schools (2.0%, n = 5/125) (Table 2). The excellent awareness of the HIV/AIDS among the participants could be due to the instituted national and international programmes that preach the *abstinence, be faithful* and condoms *use* (*ABC strategy*) message and the outcome of the study is comparable to previous studies elsewhere [31]–[35]. The ABC strategy has tremendously reduced the HIV prevalence globally and in Uganda, the strategy reduced prevalence from 30% in early 1990s to nearly 6.4% by 2004/2005 [20]–[22], but by 2011, the HIV prevalence rate has slightly increased to 7.3% possibly because of the behavioral change, knowledge gap on HIV and the socioeconomic hardship among the population thus causing a national concern in the health sector [20]–[22]. Various studies globally have reported that comprehensive school and community-based HIV prevention programs, involving provision of information on abstinence, condoms, monogamy/partner reduction, help reduce HIV prevalence rates and improve risk behaviors among young people [5] [20] [24] [25] [27] [31] [33] and this seems to have contributed to the excellent awareness of HIV/AIDS among the study participants. The finding indicated that majority of the participants were knowledgeable about the HIV/AIDs existence, though however, there were still a small group in the general population who are not aware of the HIV/AIDS existence and this group could be at a risk of acquiring the infection and possibly transmitting it to others. And therefore the message of the ABC strategy need to be further strengthened to all schools and most especially to the most vulnerable youth in the adolescent age group to which, the study findings found to be unaware of HIV/AIDS.

### 4.3. Modes of HIV/AIDS Transmission

The study findings showed that, there was variation in the responses to the modes of HIV transmission among the participants in both the rural and urban schools. The participants were able to at least mention one mode of HIV transmission. However, only 27.4% (n = 67/245) of the total participants knew all the established modes of HIV transmission [14] [32] [34] [36], with 14.3% (n = 35/120) and 13.1% (n = 32/125) from urban and rural schools respectively. The sexual intercourse with an infected person as mode of HIV transmission was the most mentioned mode (32.3%, n = 79/245) among the study participants with 18.0% (n = 44/120) and 14.3% (n = 35/125) from urban and rural schools respectively. This was followed by those who mentioned all the established modes of HIV transmission including sexual intercourse with an infected person, sharing sharp objects with an infected person, oral sex or contact through wounds with an infected person and blood transfusion from infected blood [14] [32] [34] [36]. Very few participants mentioned blood transfusion from infected blood as mode of HIV transmission (6.1%, n = 15/245) with 2.5% (n = 120) and 3.7% (n = 9/125) from urban and rural schools respectively. This was followed by sharing sharp objects with an infected person (4.4%, n = 11/245) with 2.4% (n = 6/120) and 2.0% (n = 5/125) from urban and rural schools respectively. The least mode of HIV transmission mentioned by participants was oral sex or contact through wounds with an infected person (2.4%, n = 6/245) with 0.4% (n = 1/120) and 2.0% (n = 5/125) from urban and rural schools respectively (Table 3). The findings show that, there was still knowledge gap among the participants on the awareness of all the established modes of HIV transmission including sharing sharp objects with an infected person as reported in drug abusers, oral sex or contact through wounds with an infected person and blood transfusion from infected blood [22] [27] [31] [33]. The lack of knowledge could be due to the fact that the programmes that are instituted in schools to control HIV transmission, the ABC strategy [22] [27] [31] [33] were mainly emphasizing sexual intercourse with infected person without wholesomely looking at all the established modes of HIV transmission. Whereas global comprehensive school and community-based HIV prevention programs have emphasized abstinence, condom use, as well as monogamy/partner reduction as important in reducing HIV prevalence and improve risk behaviors among young people [18] [20] [24] [25] [33] [36], they do not emphasize the delivery of all established modes of HIV transmission to the group or if provided, there is misinformation on the modes as also reported in previous studies [36]. And the lack of knowledge on these modes of HIV transmission presents a knowledge gap and could be a risk factor to the vulnerable group on HIV acquisition and prevention among the participants.

### 4.4. HIV/AIDS Prevention

The study findings showed that, majority of the participants were knowledgeable on the ABC HIV prevention strategy (83.7%, n = 205/245) with rural schools more informed about the strategy (47.4%, n = 116/125) as compared to 36.3% (n = 89/120) for urban schools. The difference could have been due to the complexity of the socioeconomic groups in urban areas where the participant reside and the exposure of these participant to sexual activities at an early age, some of whom become rebellious to their parents and thus run away from their homes to go and live on streets where they encounter different individuals with various behaviors including engaging themselves in risky sexual acts. They are also not exposed to HIV prevention programmes as compared to the rural areas. However, there was a small group of participants (16.3%, n = 40/245) with 12.7% (n = 31/120) and 3.6% (n = 9/125) from urban and rural schools respectively (Table 4), who were not knowledgeable about the ABC HIV prevention strategy and this problem was more in urban schools as compared to the rural schools. This lack of knowledge on the ABC HIV prevention strategy by the urban schools could be due to the failure of some of those schools to institute and sustain the programmes for HIV prevention that offers the continued awareness of the HIV/AIDS among the participants [5] [22] [31] [34] [36]. However, the findings showed that the participants were more knowledgeable about the ABC HIV prevention strategy as compared to all other established modes of HIV transmission [22] [27]. Therefore there is need to incorporate both the HIV prevention programmes and all the other established modes of HIV transmission wholesomely as to increase the knowledge awareness about HIV/AIDS among the participants especially the youth in the adolescent age.

### 4.5. Source of HIV Information and to Whom the Individuals’ Participants Talk to about HIV/AIDS

The study results show that the participants first got the information on HIV/AIDS as early as 4 – 8 years of age but most of them got the information about the same at the age range of 9 – 12 years (55%, 136/245) with 24.1% (n = 59/120) and 31.4% (n = 77/125) for urban and rural schools respectively (Table 5). The findings showed that participants would find comfort and prefer to talk about HIV/AIDS and sexual issues with friends and peers as compared to their parents. This was because most of the parents to the participants work and have very little time to talk to their children about the sexual matters and the HIV/AIDS while some of them are left with the maids at home who become their friends and therefore they can discuss the sexual issues and HIV/AIDS matters. Also some of their friends may have televisions as well as mobile smart phones where they can access the HIV related information on the Internet which they can pass on to their friends and hence the possible reason why friends has came out as the main source of information about HIV/AIDS to the participants.

The findings also indicate that most of the participants in both urban and rural schools got the information on HIV/AIDS and talk about it mainly from the friends (37.6%, n = 92/245) (Table 6). However, the participants also were able to get the information and talk about it from the parents, school teachers and siblings. The percentage trend on whom the participants talk to and get information on HIV/AIDS did not differ in both the urban and rural schools though it was slightly higher in rural schools as compared to urban schools except for the siblings (Table 6). This possibly could be due to the HIV/AIDS school programmes that are instituted in such schools [24] [35] that emphasizes the issues related to HIV/AIDS at all times thus making the students informed about it and its dangers.

### 4.6. Responses on Whether HIV/AIDS Is Curable

The majority of the participants, 62.0% (n = 152/245) with 30.2% (n = 74/120) and 31.8% (n = 78/125) in urban and rural areas respectively reported that there is no cure for HIV/AIDS whereas 13.1% (n = 32/245) reported that there is cure and 24.9% (n = 61/245) reported that they were not sure if it is cured. In both the urban and rural schools, there was no difference in the participants’ responses on whether HIV/AIDS can be cured or not or not sure (Table 7). The findings show that majority of the participants were aware, that HIV is not a curable disease and the drugs used only suppresses the HIV virus and boost the immune systems thus enabling the body to control the viral infection and the prevention of the opportunistic infection. The findings also show that the HIV control programmes instituted in the schools can sufficiently deliver the message that HIV infection is incurable, though some of the participants seem to be lacking such information. It is also possible that the participants were not very conversant with the term “cure” as was used in the study coupled with the misconception and myths about the cure as reported in earlier studies [36]. For those who were not sure and those that believed that there is cure for HIV/AIDS, the findings shows that there is need to be done on information delivery to the population in relation to HIV treatment and cure. The lack of updated information may be misleading and hence causing knowledge gap in management of HIV since these people can pass on false information to others especially the friends, since study findings showed that most of the participants communicate with friends mainly. However, for those whose response was *YES*, that HIV has cure, 50.0% (n = 16/32) in both urban and rural schools reported that antiretroviral (ARVs) drugs can cure it which is misleading. About 28.2% (n = 9/32) with 21.9% (n = 7/32) and 6.3% (n = 2/32) of the study participants in both urban and rural schools respectively reported that it can be cured abroad. The findings also show that 15.6% (n = 5/32) of the participants with 12.5% (n = 4/32) and 3.1% (n = 1/32) in both urban and rural schools respectively reported that it can be spiritually cured. The participants in urban schools also reported that it can be cured if transmitted to other people (6.2%, n = 2/32) (Table 8). This misconception on the belief that HIV can be cured was more in the urban schools as compared to the rural schools and a similar trend has been reported in previous studies [34] [36], and this can cause a threat in HIV management since these participants can spread such false information to other friends thus leading to HIV spread (Table 6) since many of the participant talk to their friends more than other individuals on issues related to HIV/AIDS. And this information gap in urban schools could be due to laxity or inappropriate information dissemination about HIV/AIDS amongst the youth most of whom are students.

### 4.7. Physical Recognition If Someone Has HIV/AIDS

The study findings show that 54.7% (n = 134/245) of the study participants, with 24.5% (n = 60/120) and 30.2% (n = 74/125) in urban and rural schools respectively reported that sometimes it is possible physically to recognize if someone has HIV/AIDS. And 34.3% (n = 84/245) with 19.2% (n = 47/120) and 15.1% (n = 37/125) of the participants in urban and rural schools respectively reported that it’s impossible to physically recognize if someone has HIV/AIDS. About 11.0% (n = 27/245) with 5.3% (n = 13/120) and 5.7% (n = 14/125) in urban and rural schools respectively reported that it is always possible to physically recognize if someone has HIV/AIDS (Table 9). And for the participants who reported to physically recognize if someone has HIV/AIDS, 33.5% (n = 54/161) of the participants with 20.5% (n = 33/161) and 13.0% (n = 21/161) in urban and rural schools respectively reported that such individuals are always sickly. About 31.7% (n = 51/161) with 13.7% (n = 22/161) and 18.0% (n = 29/161) in urban and rural schools respectively reported weight loss as sign of HIV/AIDS. Also 24.8% (n = 40/161) of the participants with 8.7% (n = 14/161) and 16.1% (n = 26/161) in urban and rural schools respectively reported red lips as a sign of HIV/AIDS. About 9.4% (n = 15/161) with 1.9% (n = 3/161) and 7.5% (n = 12/161) in urban and rural areas respectively reported, the presence of skin rash as sign for HIV/AIDS and 0.6% (n = 1/161) participant in urban school reported that such individuals are very rich (Table 10). Whereas most participants reported that they can physically recognize if someone has HIV/AIDS, this may not be true especially in early cases of HIV infection or HIV window period or if someone has normal CD4+ cell count which can control the opportunistic infections that in most cases are responsible for signs and symptoms reported by the participants or if someone is on antiretroviral (ARVs) drugs where the HIV infection is suppressed. In such cases, these individuals tend to look healthy with no obvious signs reported by the participants. Usually these signs occur in late stages of HIV infection and when the opportunistic infections have set, in those individuals. And this belief can be a problem among the youth especially the adolescents since they can engage in risky sexual activities with individuals who look healthy with no signs reported but infected with HIV. This facilitates the spread of HIV to others as a result of this misconception and myths as reported in earlier studies [34] [36]. The findings shows that there is a knowledge gap and belief that always HIV-infected individuals presents with the highlighted signs and symptoms thus causing a problem in controlling the HIV infection spread since the participants believe that they can tell one who has HIV/AIDS. This misconception leads to the exposure to the HIV infection with such individuals who are infected but showing no signs and symptoms of HIV infection. It is also misleading to the participants that by being rich is a sign that such a person has HIV/AIDS though in some cases, “*being rich*” sometimes is a risk factor to HIV infection since such individuals usually have multiple sexual partners hence increasing the risk of getting infected and this is a myths as reported in previous study [36]. And usually such individuals also confuse the young ladies with gifts and money, with the intention of having sexual intercourse with them and hence predisposing them to HIV infections and hence the belief by the study participants in the study. And therefore there is need to repackage the appropriate information on HIV/AIDS and emphasize some of the key issues as a way to prevent and control HIV infection from being transmitted to the youth and most especially to the adolescent group.

### 4.8. Chances of Getting HIV/AIDS

On the chances of getting HIV infection, 39.2% (n = 96/245) of the study participants with 20.4% (n = 50/120) and 18.8% (n = 46/125) in urban and rural schools respectively reported that it is impossible to get HIV infection. About 31.0% (n = 76/245) with 15.5% (n = 38/120) and 15.5% (n = 38/125) in urban and rural schools respectively reported that the chance of getting HIV infection is minimal especially if precaution are taken in relation to sexual activities with individuals who may be infected with the HIV. About 18.4% (n = 45/245) of the study participants with 8.6% (n = 21/120) and 9.8% (n = 24/125) in urban and rural schools respectively reported that the chance of getting HIV infection is high especially if someone engages themselves in a reckless life and having so many sexual partners. About 11.4% (n = 28/245) of the study participants with 4.5% (n = 11/120) and 6.9% (n = 17/125) in urban and rural schools respectively reported that the chance of getting HIV infection is moderate especially if precautions are taken by the individuals (Table 11). The findings clearly show mixed responses by the participants on possibility of acquiring HIV infection with some reporting that it’s impossible at all which is wrong perception and myths as reported somewhere else [34] [36]. Since it is known that HIV virus can be transmitted through various routes other than the sexual intercourse [5] [9] [21] [25] [37], such as in cases of accident and individuals who get wounds and then contaminated with blood of infected individuals or accidental needle pricking with infected blood in hospitals especially to health workers or sharing of sharp objects with infected individuals unknowingly especially among the youth who at times also engage themselves in alcohol and substances of abuse. Whereas the standard practices in HIV prevention, is to minimize the risk factors to HIV infection [17] [33]; however there are some inevitable circumstances like the accidents that increases one’s risk to exposure to HIV infection. This misconception and perception is a knowledge gap among the participants that need to be addressed by the relevant concerned parties’ involved in HIV control and prevention programmes.

### 4.9. View on Condom Use

On the views of condom use, 41.6% (n = 102/245) of the study participants with 20.4% (n = 50/120) and 21.2% (n = 52/125) in urban and rural schools respectively reported that condom use reduces sexual pleasure. About 34.3% (n = 84/245) participants with 18.0% (n = 44/120) and 16.3% (n = 40/125) in urban and rural areas respectively reported that using the condoms is a sign of mistrust. About 18.8% (n = 46/245) participants with 9.8% (n = 24/120) and 9.0% (n = 22/125) in urban and rural schools respectively reported that condoms are embarrassing to buy. And 2.9% (n = 7/245) participants with 0.8% (n = 2/120) and 2.1% (n = 5/125) in urban and rural schools respectively did not agree with all the above which their fellow participants had reported (Table 12). However in rural schools only, participants reported that condom use reduces sexual pleasure and it shows a sign of mistrust (1.2%, n = 3/245). And also 0.8% (n = 2/245) of the participants reported that condom use reduces sexual pleasure and they are embarrassing to buy and about 0.4% (n = 1/245) of the study participants reported that condom use is a sign of mistrust and they are embarrassing to buy (Table 12). The findings show a mixed attitude, perception and misconception on condoms and condom use among the participants which is a myth as reported earlier [36]. This shows that if the participants were to engage in sexual intercourse, majority of them are likely not to use condoms while others hate them because of various reasons similar to what has been reported in previous studies [36]. This predisposes them to HIV infection if they happen to have sex with an infected individual. The global standard on HIV prevention and control that emphasizes the ABC strategy (abstinence, being faithful and condom use) and instituting comprehensive school and community-based HIV prevention programs, that involves abstinence, condoms, as well as monogamy/partner reduction [3] [21] [22] [25] [27]. This standard has tremendously reduced the HIV prevalence in many countries around the world especially among adolescents who are school-going. The strategy emphasizes condom use and the findings from the study shows knowledge gap, misconception, poor attitude and practice on condom use. This predisposes to the vulnerable youth especially the adolescents to HIV infection if they engage themselves into risky sexual behaviors since they have misconception on condoms and condom use. Therefore there is need for organizations and the public involved in HIV prevention and control and the school programmes to emphasize to the students the relevance of each of the components of the ABC strategy including the condom use as reported in previous studies elsewhere [4] [24] [25], to ensure that the participants clearly understand them properly if the world is to reduce the HIV prevalence among the vulnerable youth especially the adolescents where the HIV prevalence is currently reported to be high and on increase in that group. Generally, the findings from this study show knowledge gap on HIV prevention, transmission and control among the youth especially in the adolescent age group thus predisposing them to HIV infection as reported in previous studies [31] [32] [35] [36].

## 5. Limitationsof the Study

The major limitation of the study was that some participants’ responses were inaccurate in relation to the question asked. Also the study was conducted during school time when students are supposed to be in class and this somehow created some inconveniences among the school staff.

## 6. Conclusion

The study findings showed that there was no gender difference among the secondary school participants in both urban and rural areas in Kampala and Buikwe districts respectively. Most of the participants were mainly Anglican, Catholics and Moslems and they had a mean age of 15.9 ± 2.5 years. Majority of the participants were aware of HIV/AIDS but there was knowledge gap on the modes of HIV transmission and prevention. The participants were aware of the ABC strategy for HIV prevention and most of the information on the HIV/AIDS was got from the friends. A sizeable number of the participants reported that HIV had no cure though some reported that it could be cured by spiritual healing or when transmitted to others and that it could be cured abroad. A number of participants reported that they could recognize one who had HIV/AIDS and they mentioned that being sickly, red lips, weight loss, skin rash and also “being very rich” were major signs. A proportion of the participants reported that they couldn’t contract HIV at all. Participants had negative attitude on condoms and condom use and they reported that condoms reduced sexual pleasures, that condom use was a sign of mistrust and that they were embarrassing to buy. This reduces the potential of participants to use condoms and hence ends up having risky sexual behaviors. Generally, participants had some knowledge on HIV/AIDS though they had knowledge gap on HIV transmission and prevention.

## Figures and Tables

**Table 1:** Demographic characteristics of participants.

Demographic characteristics	Response item	Percentage response (%)		

		Urban	Rural	Total
**Gender**	Male	24.5 (n = 60)	20.0 (n = 49)	**44.5 (n = 109)**
	Female	24.5 (n = 60)	31.0 (n = 76)	**55.5 (n = 136)**
**Total**		**49.0 (n=120)**	**51.0 (n = 125)**	**100.0 (n = 245)**
**Residence**	Participants	**49.0 (n = 120)**	**51.0 (n = 125)**	**100.0 (n = 245)**
**Religion**	Catholics	14.3 (n = 35)	14.3 (n = 35)	28.6 (n = 70)
	Anglicans	12.7 (n = 31)	18.8 (n = 46)	31.5 (n = 77)
	Pentecostals	1.6 (n = 4)	6.9 (n = 17)	8.5 (n = 21)
	Moslems	13.1 (n = 32)	8.2 (n = 20)	21.3 (n = 52)
	Others	7.3 (n = 18)	2.8 (n = 7)	10.1 (n = 25)
**Total**		**49.0 (120)**	**51.0 (n = 125)**	**100.0 (n = 245)**
**Age distribution (years)**	12 years	6.1 (n = 15)	9.4 (n = 23)	15.5 (n = 38)
	13 years	0.8 (n = 2)	0.8 (n = 2)	1.6 (n = 4)
	14 years	4.5 (n = 11)	7.8 (n = 19)	12.3 (n = 30)
	15 years	9.0 (n = 22)	7.8 (n = 19)	16.8 (n = 41)
	16 years	11.8 (n = 29)	5.7 (n = 14)	17.5 (n = 43)
	17 years	5.7 (n = 14)	5.3 (n = 13)	11.0 (n = 27)
	18 years	3.3 (n = 8)	4.9 (n = 12)	8.2 (n = 20)
	≥18 years	7.8 (n = 19)	9.3 (n = 23)	17.1 (n = 42)
**Total**		**49.0 (n=120)**	**51.0 (n = 125)**	**100.0 (n = 245)**

**Table 2:** Response on knowledge about HIV/AIDS by students in the urban and rural schools.

Response item	Response	Percentage response (%)		

		Urban schools	Rural schools	Total
**Do you know HIV/AIDS?**	Yes	46.1 (n = 113)	49.0 (n = 120)	95.1 (n = 233)
	No	2.9 (n = 7)	2.0 (n = 5)	4.9 (n = 12)
**Total**		**49.0 (n = 120)**	**51.0 (n = 125)**	**100.0 (n = 245)**

**Table 3:** Response on modes of HIV/AIDS transmission by students in the urban and rural schools.

Response item	Response	Percentage response (%)		

		Urban schools	Rural schools	Total
**How is HIV transmitted?**	1) Sexual intercourse	18.0 (n = 44)	14.3 (n = 35)	**32.3 (n = 79)**
	2) Sharing needles with infected people	2.4 (n = 6)	2.0 (n = 5)	**4.4 (n = 11)**
	3) Oral sex or wound contact with infected people	0.4 (n = 1)	2.0 (n = 5)	**2.4 (n = 6)**
	4) Blood transfusion from infected people	2.5 (n = 6)	3.7 (n = 9)	**6.1 (n = 15)**
	5) All above	14.3 (n = 35)	13.1 (n = 32)	**27.4 (n = 67)**
	6) 1 & 2	2.0 (n = 5)	2.9 (n = 7)	**4.9 (n = 12)**
	7) 1, 2 & 4	8.2 (n = 20)	11.4 (n = 28)	**19.6 (n = 48)**
	8) 1 & 4	1.2 (n = 3)	1.6 (n = 4)	**2.8 (n = 7)**
**Total**		**49.0 (n=120)**	**51.0 (n = 125)**	**100.0 (n = 245)**

**Table 4:** Response to knowledge about HIV/AIDS prevention strategy in the urban and rural schools.

	Response (Y/N)	Percentage response		Total

		Urban schools	Rural schools	
**Have you ever heard of ABC HIV prevention strategy?**	Yes	36.3 (n = 89)	47.4 (n =116)	**83.7 (n = 205)**
	No	12.7 (n = 31)	3.6 (n = 9)	**16.3 (n = 40)**
**Total**		**49.0 (n = 120)**	**51.0 (n = 125)**	**100.0 (n = 245)**

**Table 5:** Response on what age participants first got information on HIV/AIDS.

	Response age (years)	Percentage response		Total

		Urban schools	Rural schools	
**At what age did you get your first information on HIV/AIDS?**	4 – 8	6.9 (n = 17)	8.2 (n = 20)	**15.1 (n = 37)**
	9 – 12	24.1 (n = 59)	31.4 (n = 77)	**55.5 (n = 136)**
	13 – 15	15.9 (n = 39)	9.8 (n = 24)	**25.7 (n = 63)**
	16 – 20	2.0 (n = 5)	1.6 (n = 4)	**3.7 (n = 9)**
**Total**		**48.9 (n = 120)**	**51.0 (n = 125)**	**100.0 (n = 245)**

**Table 6:** Response on whom participants talk with about HIV/AIDS.

	Response age (years)	Percentage response		Total

		Urban schools	Rural schools	
**People with whom participants usually talk about HIV/AIDS**	Parents	9.0 (n = 22)	9.5 (n = 23)	**18.5 (n = 45)**
	Siblings	1.8 (n = 5)	1.0 (n = 2)	**2.8 (n = 7)**
	Friends	18.1 (n = 44)	19.5 (n = 48)	**37.6 (n = 92)**
	School teachers	7.7 (n = 19)	9.0 (n = 22)	**16.7 (n = 41)**
	All above	10.4 (n = 26)	14.0 (n = 34)	**24.4 (n = 60)**
**Total**		**47.0 (n = 116)**	**53.0 (n = 129)**	**100.0 (n = 245)**

**Table 7:** Response on whether HIV/AIDS curable.

	Response (Y/N)	Percentage response		Total

		Urban schools	Rural schools	
**Is HIV/AIDS curable**	Yes	7.3 (n = 18)	5.8 (n = 14)	**13.1 (n = 32)**
	No	30.2 (n = 74)	31.8 (n = 78)	**62.0 (n = 152)**
	Not sure	11.4 (n = 28)	13.5 (n = 33)	**24.9 (n = 61)**
**Total**		**48.9 (n = 120)**	**51.1 (n = 125)**	**100.0 (n = 245)**

**Table 8:** Response on how HIV/AIDS is cured, for those whose response were YES.

	Method of curing	Percentage response		Total

		Urban schools	Rural schools	
**How HIV/AIDS is cured, for those whose response was YES?**	ARVs cure it	15.6 (n = 5)	34.4 (n = 11)	**50.0 (n = 16)**
	Cure is abroad	21.9 (n = 7)	6.3 (n = 2)	**28.2 (n = 9)**
	Spiritual healing	12.5 (n = 4)	3.1 (n = 1)	**15.6 (n = 5)**
	Cured when transmit it	6.2 (n = 2)	0.0 (n = 0)	**6.2 (n = 2)**
**Total**		**56.2 (n = 18)**	**43.8 (n = 14)**	**100.0 (n = 32)**

**Table 9:** Response to how possible it is to recognize, physically if someone has HIV/AIDS.

	Response	Percentage response		Total

		Urban schools	Rural schools	
**Can you recognize, physically if someone has HIV/AIDS**	Yes, always	5.3 (n = 13)	5.7 (n = 14)	**11.0 (n = 27)**
	Yes sometimes	24.5 (n = 60)	30.2 (n = 74)	**54.7 (n = 134)**
	No	19.2 (n = 47)	15.1 (n = 37)	**34.3 (n = 84)**
**Total**		**49.0 (n = 120)**	**51.0 (n = 125)**	**100.0 (n = 245)**

**Table 10:** Response on how to recognize, if someone has HIV/AIDS for those whose response was YES.

	Sign for HIV	Percentage response		Total

		Urban schools	Rural schools	
**How recognize if one has HIV/AIDS, for those whose response was YES?**	Red lips	8.7 (n = 14)	16.1 (n = 26)	**24.8 (n = 40)**
	Sickly	20.5 (n = 33)	13.0 (n = 21)	**33.5 (n = 54)**
	Weight loss	13.7 (n = 22)	18.0 (n = 29)	**31.7 (n = 51)**
	Skin rash	1.9 (n = 3)	7.5 (n = 12)	**9.4 (n = 15)**
	Are very rich	0.6 (n = 1)	0.0 (n = 0)	**0.6 (n = 1)**
**Total**		**45.4 (n = 73)**	**54.6 (n = 88)**	**100.0 (n = 161)**

**Table 11:** Responses on the chances of getting HIV/AIDS.

	Response	Percentage response		Total

		Urban schools	Rural schools	
**What are the chances of you getting HIV/AIDS?**	High	8.6 (n = 21)	9.8(n = 24)	**18.4 (n = 45)**
	Moderate	4.5 (n = 11)	6.9(n = 17)	**11.4 (n = 28)**
	Minimal	15.5 (n = 38)	15.5 (n = 38)	**31.0 (n = 76)**
	Not at all	20.4 (n = 50)	18.8(n = 46)	**39.2 (n = 96)**
**Total**		**49.0 (n = 120)**	**51.0 (n = 125)**	**100.0 (n = 245)**

**Table 12:** Perception and attitude towards condoms and condom use.

	Response	Percentage response		Total

		Urban schools	Rural schools	
**Views about condoms and use**	1) Male condom reduces sexual pleasure	20.4 (n = 50)	21.2 (n = 52)	**41.6 (n = 102)**
	2) Using condoms is a sign of mistrust	18.0 (n = 44)	16.3 (n = 40)	**34.3 (n = 84)**
	3) Condoms are embarrassing to buy	9.8 (n = 24)	9.0 (n = 22)	**18.8 (n = 46)**
	4) Don’t agree with any above	0.8 (n = 2)	2.1 (n = 5)	**2.9 (n = 7)**
	5) 1 & 2 above	0.0 (n = 0)	1.2 (n = 3)	**1.2 (n = 3)**
	6) 1 & 3 above	0.0 (n = 0)	0.8 (n = 2)	**0.8 (n = 2)**
	7) 2 & 3 above	0.0 (n = 0)	0.4 (n = 1)	**0.4 (n = 1)**
**Total**		**49.0 (n = 120)**	**51.0 (n = 125)**	**100.0 (n = 245)**
